# Analysing the relationship between the fields of thermo- and electrocatalysis taking hydrogen peroxide as a case study

**DOI:** 10.1038/s41467-022-29536-6

**Published:** 2022-04-13

**Authors:** Guilherme V. Fortunato, Enrico Pizzutilo, Ioannis Katsounaros, Daniel Göhl, Richard J. Lewis, Karl J. J. Mayrhofer, Graham. J. Hutchings, Simon J. Freakley, Marc Ledendecker

**Affiliations:** 1 Institute of Chemistry of São Carlos, University of São Paulo, Avenida Trabalhador São-Carlense 400, São Carlos, SP 13566-590 Brazil; 2 Department of Interface Chemistry and Surface Engineering, Max-Planck-Institut für Eisenforschung GmbH, Max-Planck-Straße 1, 40237 Düsseldorf, Germany; 3 Forschungszentrum Jülich, Helmholtz-Institute Erlangen-Nürnberg for Renewable Energy (IEK-11), Egerlandstr. 3, 91058 Erlangen, Germany; 4 Department of Technical Chemistry, Technical University Darmstadt, Alarich-Weiss-Straße 8, 64287 Darmstadt, Germany; 5 Cardiff Catalysis Institute, School of Chemistry, Cardiff University, Main Building, Park Place, Cardiff, CF10 3AT UK; 6 Department of Chemical and Biological Engineering, Friedrich-Alexander-Universität Erlangen-Nürnberg, Egerlandstr. 3, 91058 Erlangen, Germany; 7 Department of Chemistry, University of Bath, Claverton Down, Bath, BA2 7AY UK

**Keywords:** Preventive medicine, Electrocatalysis, Heterogeneous catalysis, Sustainability, Structural properties

## Abstract

Research in thermo- and electrocatalysis have often preceded in isolation, even for similar reactions. Here, the authors compare current trends in both fields and elaborate on the commonalities and differences with a specific focus on the production of hydrogen peroxide.

## Teaser

Catalysis is inherently driven by the interaction of reactants, intermediates and formed products with the catalyst’s surface. In order to reach the desired transition state and to overcome the kinetic barrier, elevated temperatures or electrical potentials are employed to increase the rate of reaction. Despite immense efforts in the last decades, research in thermo- and electrocatalysis has often preceded in isolation, even for similar reactions. Conceptually, any heterogeneous surface process that involves changes in oxidation states, redox processes, adsorption of charged species (even as spectators) or heterolytic cleavage of small molecules should be thought of as having parallels with electrochemical processes occurring at electrified interfaces. Herein, we compare current trends in thermo- and electrocatalysis and elaborate on the commonalities and differences between both research fields, with a specific focus on the production of hydrogen peroxide as case study. We hope that interlinking both fields will be inspiring and thought-provoking, eventually creating synergies and leverage towards more efficient decentralized chemical conversion processes.

An ideal catalyst demonstrates high activity, stability, cost efficiency, and selectivity toward the targeted reaction. With the revived interest in electrocatalysis over recent years, more and more temperature-driven catalytic reactions have been targeted to be accelerated by electric potential. However, electro- and thermocatalysis have often developed in parallel rather than in collaboration, despite addressing common transformations such as oxygen reduction or hydrogen oxidation using Pt/Pd, CO_2_ reduction using Cu, alcohol oxidation using Au, or chlorine evolution/HCl oxidation on Ir and Ru oxides^1–4^. Analyzing thermal oxidation and reduction catalysis from an electrochemical view, any heterogeneous process involving changes in oxidation state, adsorption/desorption of charged species (even as spectators) or heterolytic cleavage of small molecules has parallels with electrochemistry (especially in the presence of liquid phase ionic species). In thermocatalysis, reaction temperature and pressure are used to control thermodynamic equilibrium and kinetics. In electrocatalysis, applied potential adds another parameter, with 1 V corresponding to nearly 100 kJ mol^−1^ change in free energy per electron.

Despite these parallels, few attempts have been made to bridge the gap, even for liquid phase redox processes with common materials between thermo- and electrocatalysis. As a notable exception, Spiro et al. investigated coupled oxidation-reduction reactions of ferrocyanide ([Fe(CN)_6_]^3−^+e^−^⇌[Fe(CN)_6_]^4−^) and iodide (3I^−^⇌I_3_^−^ + 2e^−^) using supported Pt catalysts^5–9^. The overall rate was determined by the exchange of electrons between the reduction and oxidation reactions through Pt nanoparticles. This suggests that slow kinetics of either redox process limits the observed rate and serves as an early example of a thermal redox reaction involving electron transfer through a catalyst. Davis demonstrated that the role of molecular O_2_ during alcohol oxidation under basic conditions is to accept electrons from the metal to regenerate hydroxide ions—akin to an electrochemical ORR reaction^10^. Conceptually this can be considered as alcohol oxidation coupled to oxygen reduction. Recent studies have suggested proton-coupled-electron-transfer (PCET) mechanisms also operate in hydrogenation reactions. The mechanism of coupled hydrogen oxidation (H_2_ ⇌ 2H^+^ + 2e^−^) and C=C/C=O reduction in liquid phase has been suggested in furanic hydrogenation via water-mediated proton transfer^11^. Free energy calculations suggested that H_2_O participates directly in the kinetically relevant reaction step, and H_2_ bypasses the direct surface reaction with the furanic derivative via a PCET.

Extending this hypothesis, it should therefore be possible to separate the active sites of oxidation and reduction if there remains a path for charge transport. In the cases cited so far, this is through a metallic nanoparticle. This conceptual approach has been achieved with immobilized enzymatic-heterogeneous catalytic systems for hydrogenation reactions where H_2_ is oxidized to H^+^ with the reduction chemistry being carried out at the co-immobilized site^12^. By considering both, oxygen reduction and hydrogen oxidation as elementary steps, the electrocatalytic and thermal synthesis of H_2_O_2_ will be used as case study to elaborate on this approach.

## Similarities in reaction mechanism between thermo- and electrocatalysis

**Fig. 1:**
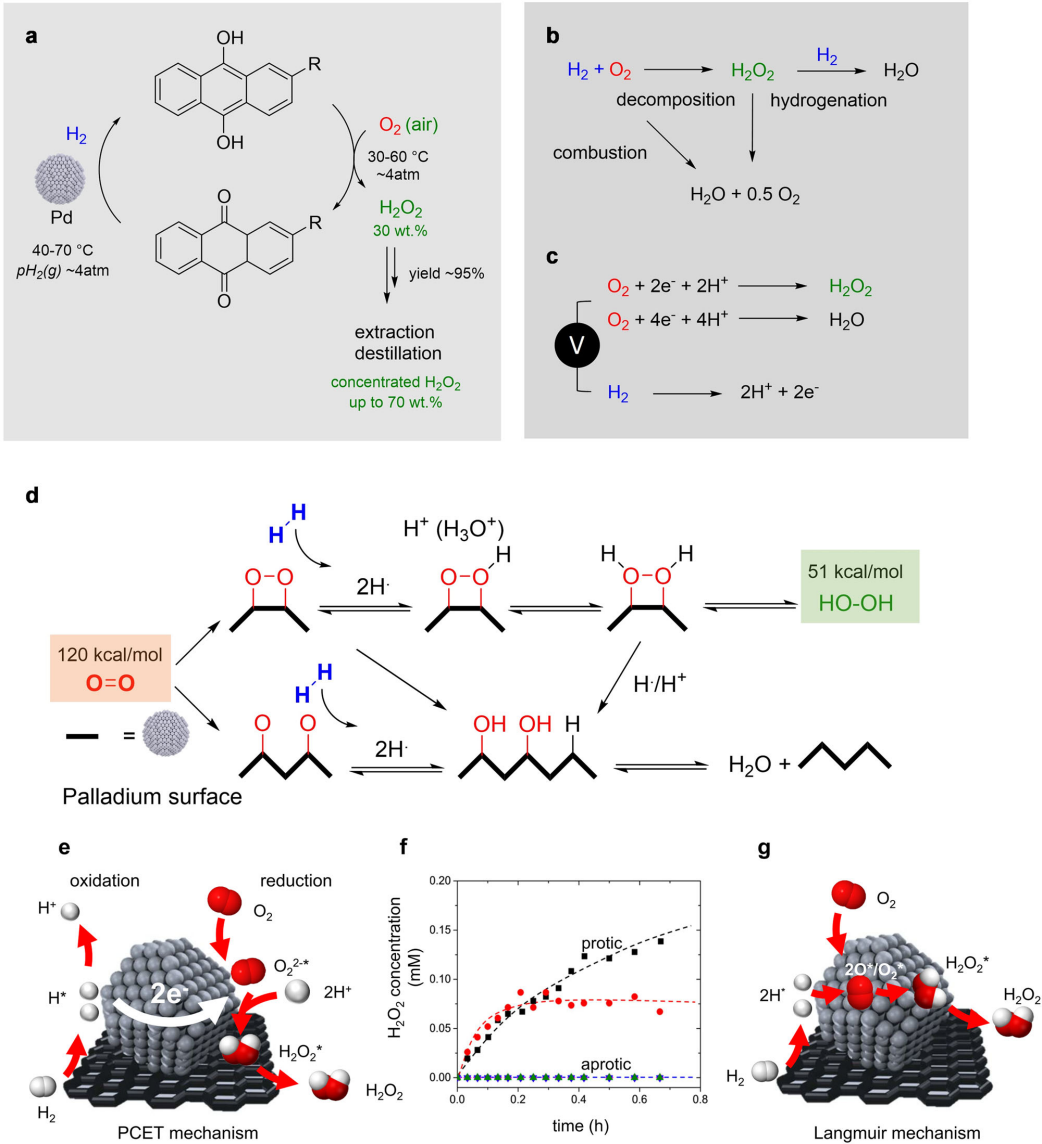
Methods to produce H_2_O_2_ Schematic of the anthraquinone process. Schematic of the anthraquinone process (**a**) direct synthesis (**b**), and electrocatalytic method (**c**) to produce H_2_O_2_ at the cathode while hydrogen is being oxidized (**d**) Scheme of the mechanism for direct synthesis of H_2_O_2_ on Pd proposed by Abate et al. Figures adapted with permission from reference^28^, Springer Nature (2006). **e** Scheme of the non-Langmuirian proposed mechanism for direct synthesis of H_2_O_2_. **f** H_2_O_2_ concentrations as functions of time during direct synthesis in protic (methanol-black and water-red) or aprotic (dimethyl sulfoxide-green, acetonitrile-blue, and propylene carbonate-magenta) media. **g** Scheme of the Langmuir mechanism for direct synthesis of H_2_O_2_. Figures adapted with permission from reference^51^, American Chemical Society (2016).

Currently, >95% of H_2_O_2_ is produced by the anthraquinone oxidation process first established in 1939 (Fig. 1a)^13^. The high capital cost results in large-scale, centralized production where highly concentrated organic working solutions are produced. Subsequently, H_2_O_2_ has to be extracted and transported over large distances after which it is diluted again and this energy is often effectively wasted^14,15^. Distributed H_2_O_2_ manufacture at the point of use is becoming an attractive alternative and would allow production to be integrated into processes permitting (i) minimal time between H_2_O_2_ synthesis and use reducing storage requirements, (ii) potential to use stabilizer-free H_2_O_2_ for more efficient oxidation processes, (iii) increased raw material security, and (iv) process intensification.

The direct reaction of H_2_ and O_2_ using thermocatalysis (direct synthesis of hydrogen peroxide, DSHP) is shown in Fig. 1b^16^. One major limitation is the precious metal content of the most efficient Pd and Pt catalysts, or their bi- and trimetallic alloys with Au^17–19^. However, the major limiting factor remains reaction selectivity to prevent water formation by direct combustion or over-hydrogenation and decomposition of H_2_O_2_. Another promising alternative is the electrochemical synthesis by a selective 2-electron oxygen reduction reaction (ORR); which is shown in Fig. 1c^20^. Similar to thermocatalysis, the design of more active and selective catalysts while decreasing the noble metal content remains a challenge. Pd catalysts dominate and the developed approaches to improve selectivity are similar in both fields. These include alloying to exploit electronic effects; site isolation to exploit geometric effects and selective surface blocking to control the concentration and orientation of surface species. Despite diverse reaction paths being possible in thermocatalytic reactions, the Langmuir–Hinshelwood mechanism is often proposed for DSHP, however, the role of charge transfer or changes in oxidation state often are not included in mechanistic steps^21–23^.

In general, higher yields of H_2_O_2_ can be achieved in acidic conditions or in the presence of protic solvents, however, little consideration is given the role of protons beyond stabilizing the formed H_2_O_2_. Choudhary was among the first to suggest a role of protons in H_2_O_2_ formation rather than surface H-atoms by publishing several studies using hydrazine in the absence of H_2_ over Pd catalysts and hydroxylamine over Au catalysts^24–26^. This demonstrated that stoichiometric reducing agents can be used in H_2_O_2_ production to supply H^+^/e^−^, drawing comparisons to electrochemical studies. Seraj et al. explicitly considered H_2_ as an electron donor in thermocatalytic nitrite reduction using AuPd catalysts, terminology that is often not considered in the discussion of thermocatalysis^27^. Abate *et al*. suggested protons hinder the breaking of the O-O bond favoring H_2_O_2_ formation over H_2_O (Fig. 1d), but also implicated protons as mechanistically relevant without consideration of similarities to electrochemical ORR/HOR^28^.

An alternative to the Langmuir model has been advanced by Wilson and Flaherty. Studying steady-state H_2_O_2_ formation rates over Pd, they found protic media was needed to produce H_2_O_2_ and that rates increased with [H^+^] drawing a definite dependence on protons in the rate-determining step. Based on this observation, the mechanism proposed is non-Langmuirian (Fig. 1e–g) where H_2_O_2_ is formed by a pathway that involves a water-mediated proton-electron transfer. More recently, Flaherty and co-workers studied the influence of redox mediators such as MeOH in the production of H_2_O_2_^29^. The authors reported that protic solvents generate surface intermediates on Pd that efficiently deliver protons and electrons to catalytic sites. The reaction was reported to consist of spatially decoupled redox reactions with a short-range electron transfer within the metal Pd catalyst. The electrons for the ORR (O_2_ + 2H^+^ + 2e^−^ ⇌ H_2_O_2_) are provided by the HOR (H_2_ ⇌ 2H^+^ + 2e^−^); thus, the two reactions can occur conceptually at two different sites provided that they are able to transfer electrons and bimetallic samples have shown consistent mechanistic features^18,30^. DFT studies on AuPd catalyst materials suggest that protonation of O_2(ads)_ from water follows a low energy route for both metals; provided a proton shuttle could be set up using two water molecules. This suggests that protonation from solvent molecules represents a reaction pathway with barriers at least as low as the direct hydrogenation from H_2_. The entire process could be considered a sequence of elementary redox processes rather than a purely surface reaction between adsorbed H and O species, consistent with the studies of Ricciardulli *et al*. on dilute PdAu catalysts with a wide range of compositions^31^. Our own groups demonstrated that single Pd-atom catalysts are able to be highly active and selective in the electrochemical 2e- ORR and HOR and the direct synthesis reaction to produce H_2_O_2_ from molecular H_2_ and O_2_^32^. Considering these liquid phase reactions as a sequence of elementary redox steps opens new approaches where physically separated half reactions with electrical connectivity can be viewed as fuel cells driven only by chemical potential. Here, the works of Yamanaka *et al*. can be interpreted as indications of this possibility. The authors described the reaction of H_2_ and O_2_ gases on physically separated electrodes to avoid mixing H_2_ and O_2_^33,34^. Here, the HOR on Pt provided a source of protons and electrons that could be directed through an external circuit to drive the ORR.

These studies suggest that (i) if electron transfer through the metal is involved, electrochemical studies of thermocatalytic materials can undoubtedly add value to our common knowledge. Furthermore, (ii) the design of new catalysts could be thought of as coupling the most effective structures for the anodic and cathodic half reaction, allowing re-evaluation of materials that have been previously discarded because of activity for only one-half reaction, and (iii) new modes of operation and concepts in material design could be possible with coupled or decoupled sites for each of the ORR and HOR half reactions.

## Deriving a common understanding

Metals such as Au that bind O_2_ or O-intermediates weakly tend to be highly selective toward H_2_O_2._ However, Au-based catalysts are associated with significant overpotentials (electrochemistry) or low H_2_ conversion (direct synthesis)^35,36^. Similarly, carbon-based materials are selective to H_2_O_2_ in the electrochemical ORR, however, are not active in heterogeneous reactions where H_2_ activation is required^37–40^. Therefore, it seems plausible that catalyst performance could be rationalized by studying the HOR/ORR for thermocatalytic materials electrochemically in isolation. Supporting this hypothesis, Flaherty and co-workers investigated Au-, Pd- and Pt-based catalysts and quantitatively demonstrated the mechanistic similarities between the direct synthesis of H_2_O_2_ and synchronous ORR and HOR electrochemistry^41^.

**Fig. 2:**
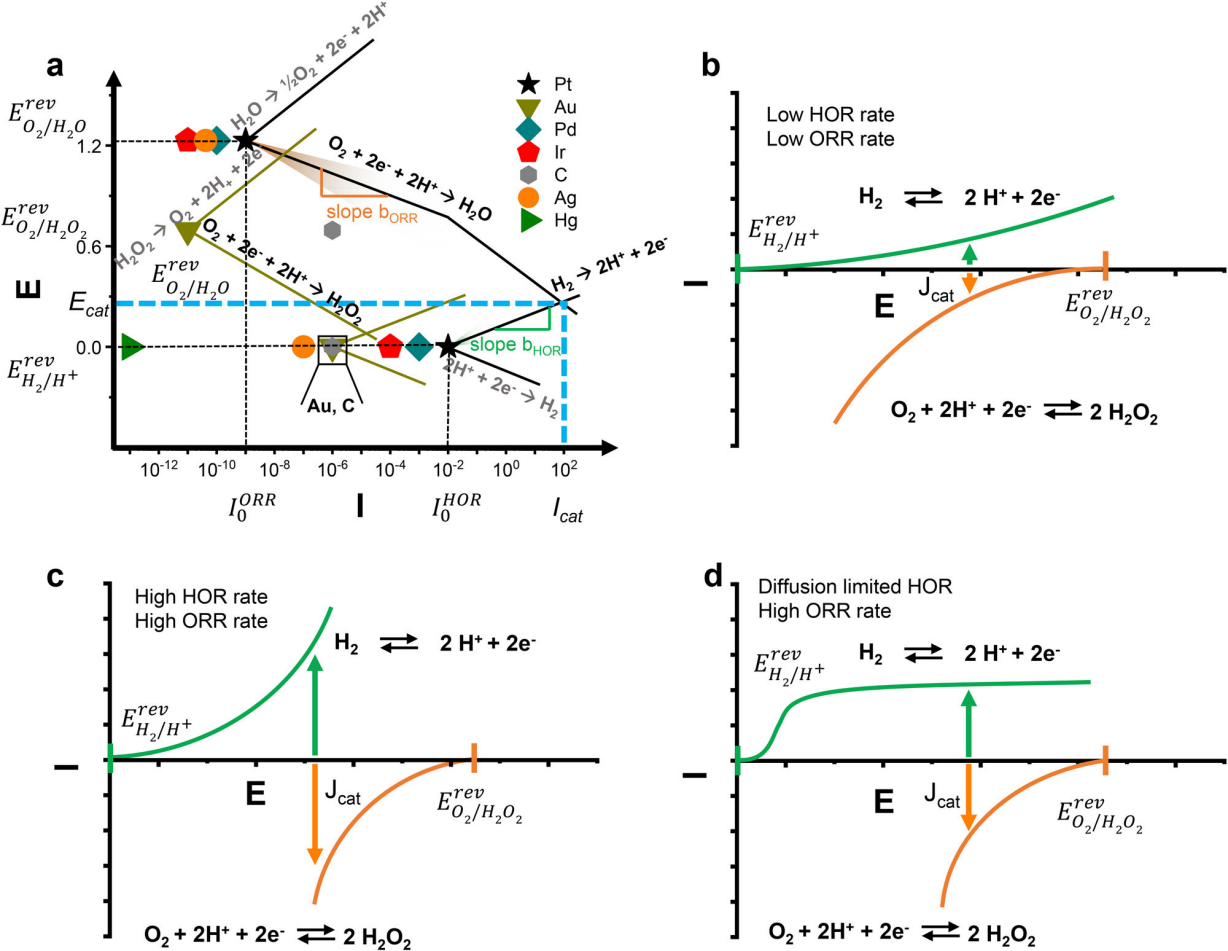
Current–voltage curves and complied Evans diagram for HOR and ORR. **a** Evans diagrams compiled from a variety of catalysts which have been reported as HOR and ORR catalysts. An exhaustive overview of all parameters can be found in Table S1. Independent current-voltage curves of both half-cell reactions with low (**b**) and high (**c**) HOR and ORR rates as well as one reaction being diffusion limited (**d**) in the framework of the mixed-potential theory.

The exchange current density, i_0_, can be defined as the rate of the forward and reverse reactions at equilibrium, analogous to a rate constant in thermocatalysis. The equilibrium potential (*E*_rev_) of each half-cell reaction can be evaluated by thermodynamic considerations while the exchange current density and the Tafel slope account for kinetic contributions. *A* large *I*_0_ indicates a high level of readiness to proceed with a certain electrochemical reaction. Together with the transfer coefficient (α), Evans diagrams can be derived for a specific catalytic reaction which represents the reaction thermodynamics (potential) and the electrode’s kinetics of a half-cell reaction. These diagrams are common in corrosion science, yet, can be readily extended to coupled redox reactions in catalysis. Evans diagrams for a variety of catalysts for electrocatalytic HOR and ORR are compiled in Fig. 2a including for Pt (black curve) and gold (ochre). The i_0_ of HER and ORR for commonly used catalytic materials vary between ~10^−13^ A cm^−2^ (Hg)^42,43^, ~10^−6^ A cm^−2^ (Au)^42,43^, ~10^−3^ A cm^−2^ (Pd)^42–44^ and ~10^−2^ A cm^−2^ (Pt)^44,45^. Data concerning non-noble metal candidates was limited due to: (i) poor corrosion resistance under acidic conditions, (ii) unfavorable intermediate adsorption strengths, or (iii) the presence of passivating surface oxides leads to low activity.

Initially, without evaluating 2e^−^ vs. 4e^−^ oxygen reduction, we considered the exchange current densities of the 4e^−^ reduction of oxygen to water. Knowing the transfer coefficient allows the exchange current at mixed potentials to be determined in analogy to galvanic processes and is defined as catalytic current density (*j*_cat_) and catalytic potential (*E*_cat_). The catalytic potential is the intermediate potential determined by the intersection of the cathodic branch (e.g., ORR) with the anodic branch (e.g., HOR) indicated by the blue line in Fig. 2a shown for Pt. At E_cat_, the rate of oxygen reduction is equal to the rate of hydrogen oxidation. Thus, if the exchange current density and the Tafel slopes of each half-cell reaction are known, it should be possible to predict the catalytic behavior in thermocatalysis.

Conceptually, the higher the catalytic current density between the coupled HOR–ORR redox reactions at relevant conditions, the higher the maximum rate achievable in DSHP reaction (considering overall O_2_ reduction to both H_2_O_2_ and H_2_O). The reaction rate is therefore limited by the smallest contribution to the ORR/HOR couple. As a benchmark, TOF (h^−1^) and yields (mol kg^−1^ h^−1^) for the selected heterogeneous catalysts are shown in Supplementary Table 1. Intriguingly, materials such as Au or Ag possess relatively low catalytic current densities as the half-cell kinetics for the HOR are poor (hypothetically shown in Fig. 2b)^41^. However, due to low H_2_O_2_ residence time and the subsequent hindrance of oxygen adsorption, they tend to demonstrate high selectivity. Despite the relatively low ORR exchange current densities of Au, the reported TOFs are often comparable to metals such as Pt in reactions without acid and halide additives which have larger exchange currents (hypothetically shown in Fig. 2c) but likely for different reasons (Au—low conversion and high selectivity, Pt—high conversion low selectivity). The reason for this difference can also be qualitatively explained from existing electrochemical studies. Over hydrogenation and H_2_O_2_ decomposition takes place due to the high activity for H_2_ activation or HOR on Pt compared to Au coupled with strong binding of O-intermediates. The comparison of existing materials hints strongly to the possibility that catalysts can be evaluated separately in half-cells through quick, electrochemical screening and suitable material combinations with electronic connectivity can be combined. However, limitations in comparability between thermo- and electrocatalysis must also be considered.

## Limits in the comparability

The presumably greatest limitation of comparing both fields lies in their different reaction environments. Most electrochemical approaches are based on using standard conditions for temperature (298.15 K) and pressure (1 bar) in the presence of a conductive and acidic electrolyte with typically low rates of reactant diffusion. High reaction rates in the direct synthesis are also typically limited by reactant solubility in aqueous or alcohol/aqueous based solvent systems. For thermocatalytic systems, working pressures up to 40 bar are common (containing H_2_ and O_2_ mixtures in a diluent such as N_2_ or CO_2_) and sub-ambient temperatures are often utilized especially in the absence of acid or halide additives to stabilize H_2_O_2_.

The exchange current density i_0_ also strongly depends on the solution composition adjacent to the electrode’s surface and can be expressed by Eq. 1.

1 $${{i}}_{0({{{{{\rm{T}}}}}},{{{{{{\rm{C}}}}}}}_{{{{{\rm{O}}}}}},{{{{{{\rm{C}}}}}}}_{{{{{\rm{R}}}}}})}={{{{{{\rm{i}}}}}}}_{0\left({{{{{{\rm{T}}}}}}}^{* },{{{{{{\rm{C}}}}}}}_{{{{{\rm{O}}}}}}^{* },{{{{{{\rm{C}}}}}}}_{{{{{\rm{R}}}}}}^{* }\right)}{\left(\frac{{C}_{{{{{\rm{R}}}}}}}{{C}_{{{{{\rm{R}}}}}}^{* }}\right)}^{\gamma }\,{\left(\frac{{C}_{{{{{\rm{O}}}}}}}{{C}_{{{{{\rm{O}}}}}}^{* }}\right)}^{\delta }{e}^{\frac{{-E}_{{{{{{\rm{act}}}}}}}}{{RT}}}$$

${i}_{0}\left({{{{{\rm{T}}}}}}^{*},{{{{{\rm{C}}}}}}_{{{{{\rm{O}}}}}}^{* },{{{{{{\rm{C}}}}}}}_{{{{{\rm{R}}}}}}^{* }\right)$ [A ${{{{{{\rm{cm}}}}}}}_{{{{{{\rm{real}}}}}}}^{-2}$] as exchange current density at defined reference temperature and reference reactant/product $({{{{{\rm{C}}}}}}_{{{{{\rm{O}}}}}}^{* },\,{{{{{{\rm{C}}}}}}}_{{{{{\rm{R}}}}}}^{* })$ concentrations, γ, δ as reaction order and *E*_act_ as activation energy of the exchange current density^46^. From Eq. 1, the temperature and the surface concentrations can have drastic effects on *i*_0_. Butler-Volmer kinetics dictate a linear relationship of Tafel slope (b) and temperature, however, a linear dependency of *b* and *T* is not always found^47^. The reasons for this have been reported to range from the expansion of the inner region of the double layer with temperature, variable temperature-dependent adsorption of spectator species, structural solvent changes, or to altered solvation spheres around reactant ions^46^. Additionally, diffusion limitation might be depicted as concentration polarization exemplarily shown in Fig. 2d for the HOR. Here, the reaction of H_2_ at large overpotentials may cause a depletion of gas and the transport of H_2_ to the catalyst’s surface becomes diffusion limited resulting in the shown concentration polarization, effecting directly *i*_cat_ and *E*_cat_. In principle, these limitations could also be measured by electrochemical means. However, in order to allow useful correlation between both fields, interfacial effects in electrocatalysis such as oriented water dipoles, present ions, non-covalent intermediate/water interactions, and the electric field have to be comparably small and Sabatier’s principle (binding intermediates not too strongly and not too weakly) dominates the reaction mechanism. This is often the case as shown by modeling electrochemical processes by conducting gas-phase calculations on well-defined catalytic surfaces^48–50^.

## Conclusion

Overall, comparing both fields, the thermo- and electrocatalytic H_2_O_2_ production reveal many similarities. For both reactions, the catalyst selection narrows down to similar materials, mainly Pd and Pt-based materials. Intriguingly, more materials for the electrocatalytic ORR have been reported that do not appear in the thermocatalytic synthesis of H_2_O_2_. The reason might lie in a non-Langmuirian reaction mechanism where spatially decoupled redox reactions take place. Specifically, the ORR acts as electron sink provided for the hydrogen oxidation reaction when both are electronically connected. These observations are supported by the compilation of potential–current density diagrams that can be used to bridge the gap between both fields. Moreover, the selection of catalyst material is generally dictated by activity considerations only, while strategies to increase the selectivity are often lacking. Further enhancement of the overall performance will greatly rely on the joint optimization of both catalytic properties, eventually guided by the investigations of half-reactions via electrochemical methods. In general, this approach of combining know-how from thermo- and electrocatalysis that has been laid out for the example of H_2_O_2_ synthesis can be adapted to a wide range of reactions including oxidation and reduction processes at interfaces. Developments along this path can be mutually beneficial, leading to more efficient chemical technologies and novel applications.

## Supplementary information

Supplementary Information
