# Supplementary material for: Analysing the relationship between the fields of thermo- and electrocatalysis taking hydrogen peroxide as a case study
Source: Nat Commun. 2022 Apr 13;13:1973. doi: 10.1038/s41467-022-29536-6 (PMC9007970; doi:10.1038/s41467-022-29536-6)
Supplement: Supplementary file 1 — Supplementary Information [file 41467_2022_29536_MOESM1_ESM.pdf]

Supplementary information for

Analysing the relationship between the fields of thermo- and electrocatalysis taking hydrogen peroxide as a case study

Guilherme V. Fortunato[1], Enrico Pizzutilo[2], Ioannis Katsounaros[3], Daniel Göhl[4], Richard J. Lewis[5], Karl J. J. Mayrhofer[2,3,6], Graham. J. Hutchings[5], Simon J. Freakley[7]* and Marc Ledendecker[4]*

1) Institute of Chemistry of São Carlos, University of São Paulo, Avenida Trabalhador São-Carlense 400, São Carlos, SP 13566-590, Brazil

2) Department of Interface Chemistry and Surface Engineering, Max-Planck-Institut für Eisenforschung GmbH, Max-Planck-Straße 1, 40237 Düsseldorf, Germany

3) Forschungszentrum Jülich, Helmholtz-Institute Erlangen-Nürnberg for Renewable Energy (IEK-11), Egerlandstr. 3, 91058 Erlangen, Germany

4) Department of Technical Chemistry, Technical University Darmstadt, Alarich-Weiss-Straße 8, 64287 Darmstadt, Germany

5) Cardiff Catalysis Institute, School of Chemistry, Cardiff University, Main Building, Park Place, Cardiff, CF10 3AT, UK.

6) Department of Chemical and Biological Engineering, Friedrich-Alexander-Universität Erlangen-Nürnberg, Egerlandstr. 3, 91058 Erlangen, Germany

7) Department of Chemistry, University of Bath, Claverton Down, Bath BA2 7AY, UK

Contents

1. Supplementary Table 2
2. Supplementary Figure 3
3. Supplementary References 4

1. Supplementary Table

Supplementary Table 1. Exchange current density (*I_0*), Tafel slopes (b) for HOR and ORR in acidic medium, E_{cat} and I_{cat} (values obtained from Evans diagrams in Supplementary Figure S1); turnover frequency (*TOF*), activity, and selectivity for the thermocatalytic synthesis of H_2O_2 for a variety of material classes.

	**Electrocatalysis**							**Thermocatalysis**					
**Catalyst**	***I_0* HOR (A cm^{-2})**	***b_{HOR}* (mV dec^{-1})**	***I_0* ORR (A cm^{-2})**	***b_{ORR}* (mV dec^{-1})**	***E_{cat}* (V)**	***I_{cat}* (A cm^{-2})**	**Ref.**	***TOF* (h^{-1})**	**Productivity (mol kg^{-1} h^{-1}) [a]**	**Conversion (%)**	**Selectivity (%)**	**H_2O_2 Decomposed (%) [b]**	**Ref.**
**Pt**	$1.0x10^{-2}$	30	$1.0x10^{-9}$	60	0.27	82	[1-4]		5 [c]	4 [c]	16 [c]	7 [c]	-
**Pd**	$1.0x10^{-3}$	110	$1.0x10^{-10}$	60	0.35	1.5	[1,3,5,6]	399 [d]	23 [c]	21 [c]	32 [c]	71 [c]	[7]
**Ir**	$1.0x10^{-4}$	122	$1.0x10^{-11}$	60	0.37	0.13	[8-12]		n.d [c]	n.d [c]	1 [c]	1 [c]	-
**Au**	$1.0x10^{-6}$	116	$1.0x10^{-11}$	120	0.05	$2.4x10^{-6}$	[3,5,6,13]	8.3 [e]	4 [c]	75 [c]	12 [c]	0 [c]	[14]
**C**	$1.0x10^{-6}$	206*	$1.0x10^{-6}$*	110	0.45	$1.4x10^{-4}$	[10,15,16]	Not evaluated for DSHP.					-
**Ag**	$1.0x10^{-7}$	60	$4.2x10^{-11}$	112	0.11	$6.7x10^{-6}$	[4-6,17-19]	0 [f]	n.d [c]	1 [c]	1 [c]	n.d [c]	[20]
**Hg**	$1.0x10^{-13}$	114	-	-	-	-	[5,6,21]	Not evaluated for DSHP.					-

*Values obtained in alkaline medium.

- Values not available.

[a] H_2O_2 direct synthesis reaction conditions: Catalyst (0.01 g), H_2O (2.9 g), MeOH (5.6 g), 5% H_2/CO_2 (420 psi), 25% O_2/CO_2 (160 psi), 0.5 h, 2° C, 1200 rpm.

[b] H_2O_2 degradation reaction conditions: Catalyst (0.01 g), H_2O_2 (50 wt.% 0.68 g) H_2O (2.22 g), MeOH (5.6 g), 5% H_2/CO_2 (420 psi), 0.5 h, 2°C, 1200 rpm.

[c] New experimental data obtained for $Pt_{1\%}$/ C, $Pd_{1\%}$/ C, $Ir_{1\%}$/ C, $Au_{1\%}$/ C, and $Ag_{1\%}$/ C catalysts.

nd = not detected

[d] $Pd_{3\%}$/ SiO_2, 0.1 MPa, 60 mL min^{-1}, 0.5 h, 10 °C, ethanol solvent.

[e] $Au_{5\%}$/ C, 4.0 MPa, 0.5 h, 2 °C, methanol/water solvent.

[f] $Ag_{1\%}$/ C, 3.0 MPa, 0.25 h, 2 °C, 0.03 M H_2SO_4 methanolic solution.

2. Supplementary Figure

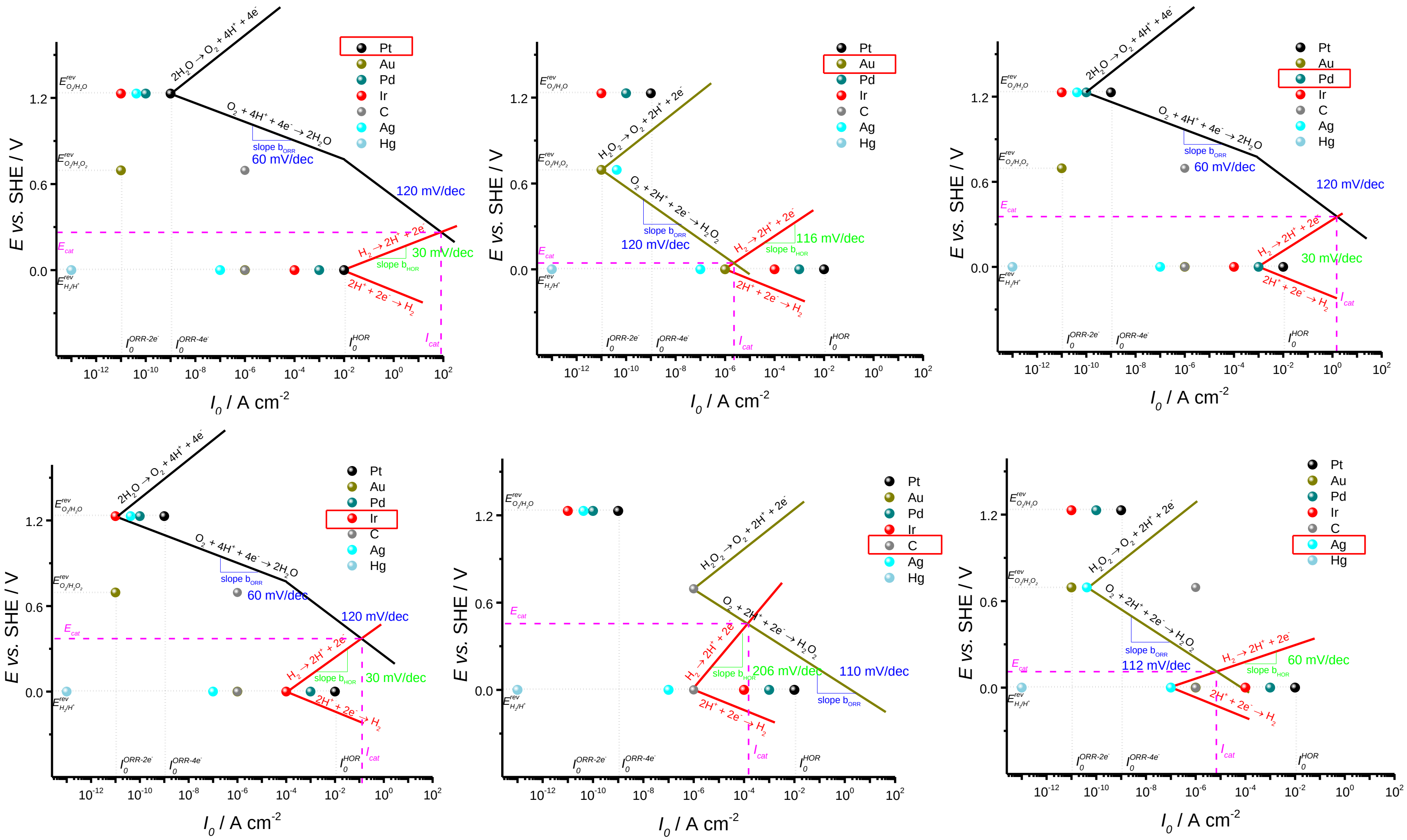

Supplementary Figure 1. Evans diagrams compiled from a variety of material classes have been reported as HOR and ORR catalysts.

3. Supplementary References

1 Durst, J. *et al.* New insights into the electrochemical hydrogen oxidation and evolution reaction mechanism. *Energy Environ. Sci.* **7**, 2255-2260 (2014).

2 Neyerlin, K., Gu, W., Jorne, J. & Gasteiger, H. Study of the exchange current density for the hydrogen oxidation and evolution reactions. *J. Electrochem. Soc.* **154**, B631-B635 (2007).

3 Damjanovic, A. & Brusić, V. Oxygen reduction at Pt-Au and Pd-Au alloy electrodes in acid solution. *Electrochim. Acta* **12**, 1171-1184 (1967).

4 Bockris, J. O. M., Ammar, I. A. & Huq, A. K. M. S. The mechanism of the hydrogen evolution reaction on platinum, silver and tungsten surfaces in acid solutions. *J. Phys. Chem.* **61**, 879-886 (1957).

5 Trasatti, S. Work function, electronegativity, and electrochemical behaviour of metals. *J. Electroanal. Chem. Interf. Electrochem.* **39**, 163-184 (1972).

6 Conway, B. E. & Tilak, B. V. Interfacial processes involving electrocatalytic evolution and oxidation of H_2, and the role of chemisorbed H. *Electrochim. Acta* **47**, 3571-3594 (2002).

7 Ouyang, L. *et al.* Functionalized silica nanorattles hosting Au nanocatalyst for direct synthesis of H_2O_2. *Catal. Today* **248**, 28-34 (2015).

8 O'Hayre, R., Cha, S.-W., Colella, W. & Prinz, F. B. *Fuel Cell Fundamentals*. (John Wiley & Sons, Inc, 2016).

9 Gnanamuthu, D. S. & Petrocelli, J. V. A generalized expression for the Tafel slope and the kinetics of oxygen reduction on noble metals and alloys. *J. Electrochem. Soc.* **114**, 1036 (1967).

10 Shinagawa, T., Garcia-Esparza, A. T. & Takanabe, K. Insight on Tafel slopes from a microkinetic analysis of aqueous electrocatalysis for energy conversion. *Sci. Rep.* **5**, 13801 (2015).

11 Sepa, D. B., Vojnovic, M. V., Stojanovic, M. & Damjanovic, A. Kinetics of oxygen reduction at iridium electrodes in aqueous solutions. *J. Electroanal. Chem. Interf. Electrochem.* **218**, 265-272 (1987).

12 Durst, J., Simon, C., Hasché, F. & Gasteiger, H. A. Hydrogen oxidation and evolution reaction kinetics on carbon supported Pt, Ir, Rh, and Pd electrocatalysts in acidic media. *J. Electrochem. Soc.* **162**, F190-F203 (2014).

13 Adić, R. R., Marković, N. M. & Vešović, V. B. Structural effects in electrocatalysis: Oxygen reduction on the Au (100) single crystal electrode. *J. Electroanal. Chem. Interf. Electrochem.* **165**, 105-120 (1984).

14 Edwards, J. K. *et al.* Switching Off hydrogen peroxide hydrogenation in the direct synthesis process. *Science.* **323**, 1037-1041 (2009).

15 Yue, X. *et al.* $K_{0.4}TaO_{2.4}F_{0.6}$ Nanocubes as highly efficient noble metal-free electrocatalysts for hydrogen evolution reaction in acidic media. *Electrochim. Acta* **245**, 193-200 (2017).

16 Zhang, Q. *et al.* Highly efficient electrosynthesis of hydrogen peroxide on a superhydrophobic three-phase interface by natural air diffusion. *Nat. Commun.* **11**, 1731-1731 (2020).

17 Blizanac, B. B., Ross, P. N. & Markovic, N. M. Oxygen electroreduction on Ag(111): The pH effect. *Electrochim. Acta* **52**, 2264-2271 (2007).

18 Petrii, O. A. & Tsirlina, G. A. Electrocatalytic activity prediction for hydrogen electrode reaction: intuition, art, science. *Electrochim. Acta* **39**, 1739-1747 (1994).

19 Sleightholme, A. E. S., Varcoe, J. R. & Kucernak, A. R. Oxygen reduction at the silver/hydroxide-exchange membrane interface. *Electrochem. Commun.* **10**, 151-155 (2008).

20 Gu, J., Wang, S., He, Z., Han, Y. & Zhang, J. Direct synthesis of hydrogen peroxide from hydrogen and oxygen over activated-carbon-supported Pd–Ag alloy catalysts. *Catal. Sci. Technol.* **6**, 809-817 (2016).

21 Conway, B. E. & Bockris, J. O. M. Electrolytic hydrogen evolution kinetics and its relation to the electronic and adsorptive properties of the metal. *J. Chem. Phys.* **26**, 532-54 (1957).
